# La Charite

**Published:** 1829-10-01

**Authors:** 


### HOPITAL DE LA CHARITE.

### Diseases of the Prostate Gland and Vesiculæ Seminales. Affection of the Head.*

M. Dalmas, one of the rédacteurs of our

---

* Journ. Hebd. No. 33.

French hebdomadal contemporary, has published a not uninteresting paper or report under the above title. The object of the author will be best explained by detailing his cases first, and then the deductions which he draws from them. We may premise, however, that M. Lallemand, in his work on the Diseases of the Urethra, has remarked on the similarity between some affections of the generative organs and those of the brain and spinal marrow, a similarity which that celebrated professor believes to be productive of numerous errors in practice. Upon this hint it seems M. Dalmas writes, and we therefore proceed without further comment to his cases.

*Case* 1. This is not our author's own, but taken from Stoll.

A musician, ætatis 32, was admitted into Hospital on the 8th October, with delirium, and small, weak, and very rapid pulse. The only account that could be got of him was, that he had been confined to bed for a month, during which time a surgeon had been treating him for disease of the testicles. On admission, the prepuce was in a callous state, and immoveably adherent to the glans, which was left half uncovered. The left testicle appeared to be enlarged, and the orifice of the urethra was so narrow that no bougie could be made to enter it. Blisters, sinapisms, infusions of serpentaria, and contrajerva, with camphor, in large doses, had the effect of raising the force of the pulse, and, at the expiration of two days, the patient recovered his senses sufficiently to state that, after repeated gonorrhœas, he had not been able, for the last eight years, to make water without the greatest difficulty and distress. He was about to mention some more particulars, when he fell again into his former delirious state, and died on the 12th. During his continuance in hospital he scarcely voided any urine, but at length an instrument was passed, and the contents of the bladder discharged.

*Dissection.* Near the frænum of the prepuce was a stricture of the urethra, but beyond that point the canal was perfectly sound. The orifices of the ejaculatory ducts were dilated, and pus issued from the left on compressing the prostate. Pus was also squeezed out from all the excretory ducts of the latter gland, and on cutting it open, a number of little abscesses were found on it, some the size of a lentil, and others that of a pea. The left vesicula seminalis was filled with pus, and its coats hard, thickened, and inflamed in several points. The tunica vaginalis was closely adherent to the left testicle, which was larger than the right, and contained in its substance an abscess as large as a nut. The bladder contained a little bloody urine, and its mucous membrane presented large patches of deep red colour, looked like marks of contusion. The kidneys were redder than usual; the intestines were inflamed.

The left lung was filled with crude tubercles, and the intervening parenchyma was altered in structure. The pleuræ on this side were adherent, considerably thickened, and formed into laminæ separable one from the other. The lateral ventricles of the brain were full of yellow serum.

*Case* 2. Jean Pica, ætatis 24, a servant and unmarried, was admitted into the Charité on the 14th November, 1827, with hot skin, flushed face, dry tongue, pulse 98, small and sharp, restlessness, and pain in the head. The bowels had not been opened for two days, and he had occasional cough, but the respiration was heard well throughout the chest. Two months previously he had had what appeared to have been inflammation of the peritoneum in the hypogastric region, which was cured by antiphlogistic measures. The cough had existed for six weeks, the symptoms for which he was admitted were only of four days duration. A tisane only was prescribed; next day the patient was worse, the belly being painful upon pressure, and his answers unconnected. Fifteen leeches to the basis cranii, oxymel drink, sinapisms to the lower extremities, and lavements, were had recourse to, but on the 16th the delirium was more marked, the tongue was dry, and convulsive actions of the muscles, which the patient had previously had, were now in great measure replaced by subsultus tendinum. A vein was opened in the arm, the delirium increased, and on the 19th, the pupils were dilated, with retention of urine. These symptoms augmented, and in the evening of the 20th, the patient died.

*Dissection in the morning of the 22d.* The membranes of the brain were dry, the convolutions flattened, the ventricles filled with serous fluid of a milky colour, and the septum lucidum softened. Both lungs presented miliary tubercles, but no vomica or cavern. Two folds of the small intestines were bound together by recent false membranes. The mucous membrane of the bladder was of dark red colour, studded with some patches of lymph, and thickened. The cavity of the bladder was half filled with turbid urine. The prostate was slightly enlarged, and on squeezing it a quantity of yellowish matter issued from the excretory ducts. On making the section of the gland the appearance, both to M.M. Dalmas and Lallemand, was that of suppuration in the follicles. The left vesicula seminalis was double the volume of the right, of deep yellow colour, and in its cavity, which had lost in great degree the septa it naturally presents, "veritable purulent matter" was contained. The vas deferens on this side was obstructed for the space of five or six inches by matter of the same description but of greater consistence. The mucous follicles of the inner membrane were also filled with it.

This patient, it should be stated, had never laboured under any gonorrhœal or syphilitic affection.

*Case* 3. Michel Boex, æt. 23, a journeyman, was admitted into hospital, September 10th, under the care of M. Rullier, in a remarkable state of fatuity and depression. He could give no satisfactory account of himself, but those who brought him said that he had laboured, for three months, under diarrhœa, which was lately checked, and that he vomited every thing he took; the pulse was not above 60 at the most. M. Rullier, struck with the state of stupor and depression in which the patient was, ordered sinapisms and emollients. The skin grew slowly warm, the pulse rose, and the stupor changed into moderate delirium, with subsultus tendinum and involuntary discharge of the urine. Leeches, blister to the neck, infusion of arnica, and a lavement, were the means employed; but, on the 17th, we find dilatation of the pupils, and coma interrupted by restlessness and expression of complaint. Next morning death took place.

*Dissection.* The arachnoid tunic was "troubled" on the anterior surface of the hemispheres of the brain; the pia mater infiltrated here and there with sero-purulent fluid; the substance of the brain injected in a high degree; the septum lucidum softened, but not entirely disorganized. The mucous membrane of the stomach was of reddish brown colour, and softened in the greater part of its extent, whilst in several places were small round ulcers apparently confined exclusively to the tunic in question. The great intestine was the seat of chronic inflammation, and exhibited its consequences, swelling, induration, injection, and numerous ulcers of the mucous membrane. The urethra was healthy, so was the bladder, but, on squeezing the prostate, pus, or rather a pultaceous matter, was expressed. The vesiculæ seminales were filled with the same sort of substance, and so were the vasa deferentia.

*Case* 4. This patient was not seen by M. Dalmas during life, but he was informed by M. Littré, who conducted the post-mortem examination, that he died with head symptoms, and that traces of meningeal inflammation were found upon dissection.

The bladder was filled with limpid urine, and a stricture of the urethra existed at the distance of an inch and a half from the neck of the bladder. The left half of the prostate was "scirrhous," the right was whiter than natural. The vesiculæ siminales were small, hard, and contained a dirty-looking matter in very small quantity. The vasa deferentia, from circumstances, could not be examined, except in that part of their course which intervenes between the testicle and the outer abdominal ring. They presented puriform swellings, increasing in volume the nearer they were to the testis, and containing soft cheesy looking matter. The cavity of each vas deferens between these enlargements was obliterated, and near the testicles the cheesy matter was replaced by more genuine-looking pus. The left epididymis was converted into an irregular tumour, as large as the testicle itself, and the corpus Highmorianum of the latter was "evidently scirrhous." The right testicle was tolerably sound, but the epididymis was dwindled into a small cylindrical cord, and where it becomes vas deferens, one of the swellings described on the latter communicated with a fistulous opening in the scrotum. Another scrotal fistula opened into the corpus Highmorianum. Each tunica vaginalis presented specimens of encysted hydrocele.

So much for the facts on which our author proceeds to build some reasonings; and now let us look for a moment at the latter. He believes that all four cases were examples of the same affection, and that the matter discovered in the vesicula seminalis of one, and the prostate, vas deferens, or even epididymis of the other, presented too much similarity of physical character, to be other than identical. The differences of density, &c. he believes to be easily explained by the difference of organs in which it was found, and the period of its formation. Another question mooted by M. Dalmas is the origin of the matter, and this he thinks to have been simple inflammation, rather than any tuberculous diathesis. The grounds for his opinion we need not detail, as they are bottomed on the facts of the case themselves. M. Dalmas believes the prostate gland to be first affected, then the vesiculæ seminales, next the vasa deferentia, and, finally, the epididymis and testicle itself. The minutiæ of his theory, for theory it is after all, we omit, in order to notice the sympathetic affection of the nervous system produced by this disease of the genital apparatus. Our author observes, that we may suspect a morbid condition of these organs, when, in persons about the proper age, inflammatory affection of the abdomen is followed after an interval by delirium, fever without paralysis or convulsion, retention of urine, and pain in the hypogastrium; especially if these symptoms have been preceded by gonorrhœa, discharges, or swelling of the testicle.

So much for the paper of M. Dalmas, which is certainly possessed of much interest, if not novelty. We candidly confess, however, that his cases seem to us more important than his inferences, and likely to prove of service, whether the latter be right or wrong. The space to which we limit our Periscopic articles, prevents us from making some remarks on these cases at present, but no doubt a future opportunity will occur for indulging in our rights as reviewers. We would simply remark, that we think it by no means proven that inflammation existed in all, and that therefore the investigation is open to unbiassed and diligent enquirers. Neither is it so certain, in our humble opinion, that the fons et origo of the fatal symptoms was unequivocally located in the genital system, at least in all of the cases in question. However, our knowledge of the affections of the prostate, and particularly of the vesiculæ seminales, is yet so completely in its infancy, that dogmatical opinions on the subject would be both presumptuous and absurd.

### II. PLEURITIS HÆMORRHAGICA—OPERATION FOR EMPYEMA—DEATH—DISSECTION. Wards of M. LERMINIER. Reported by M. PORTAL.

*Case.* Dominique Lyon, aged 28 years, entered LA CHARITÉ on the 17th Feb. 1829, having been ill about three weeks, but not incapable of working at his business of jeweller. He stated that, for six weeks previously, he had had wandering pains in the left side of the chest, aggravated by damp weather and by corporeal exertion. A fortnight before he entered the hospital, the pain in his side became acute, accompanied by cough, without expectoration, but attended with dyspnœa, especially on ascending stairs. To these symptoms were added debility and prostration.

No medical means had been employed. On examination at the hospital, (18th February) the following symptoms were noted:—considerable dyspnœa — frequent cough, with trifling expectoration —some pain in the left side of the thorax—pulse 90. Auscultation shewed bronchial respiration throughout almost the whole of the posterior part of the left lung — but no respiration in the front and inferior portions of the same. In the upper and front part the breathing was louder than natural (puerile.) The sound, on percussion, corresponded with the auscultic indications. The patient was copiously bled four times in succession, without any apparent benefit, and diuretic medicines were exhibited.

On the 21st of February, the dyspnœa was found to be much increased—the cough frequent—the dulness of sound in the left side was now complete, both before and behind. Egophony was heard at the very summit of the chest. The left side, on being measured, was found to be an inch larger than the right side. The intercostal spaces were also wider. The heart was found to be displaced, and the pulsations were felt in the right side of the chest, five or six inches from the natural situation.

On the 12th of March, the symptoms were so pressing, that life was threatened, and next day M. Roux was called in. The presence of a fluid in the left side of the chest was ascertained, and the distiguished surgeon above-named, performed the operation for empyema in the usual place. When the stilet was withdrawn a very sanguinolent fluid flowed out freely, for a few seconds, but then stopped, except when the patient coughed. Air rushed into the chest at each deep inspiration.* Not more than ten or twelve ounces of sanguinolent fluid were obtained; but even this evacuation appeared to give considerable increase of facility to the breathing. But this amelioration only lasted about an hour, when the pulse was found to be 120—the anxiety great—and the patient unable to lie on any but the left side. He died the next day, 14th March.

*Dissection.* A small puncture being made into the left side of the thorax, and a candle being held opposite the puncture, an inflammable gas issued forth and became ignited for some seconds, and disengaged an odour of sulphuretted hydrogen. On making a larger opening there issued three or four pints of a very sanguinolent fluid. The costal and pulmonary pleuræ were found covered with false membranes, some of them more than two lines in thickness. In the substance of some of these false membranes, and even in the substance of the pleura, numerous small tubercles were discovered. Part of the right lung was hepatized. The heart was pushed to the right of the middle line of the sternum. The pericardium contained a considerable quantity of fluid blood, without any clots. There were a good number of small ulcerations in the mucous membrane of the stomach—no other lesions of any consequence in any of the organs.

*Remarks.* It was doubted by some of the medical men present at this dissection, whether the sanguinolent fluid found in the left side of the chest was not the result of some hæmorrhage from the operation tinging the serous fluid previously in that side. The reporter, M. Portal, rejects this opinion, and insists that it was a *sanguinolent effusion*, —or, in other words, an hæmorrhagic pleurisy—averring also, that the blood found in the pericardium had traversed the parietes of that bag, from the general cavity of the pleura, "par cette singuliere propriété qu'ont les tissus vivans de transmettre à travers leurs trames les liquides, &c." We do not see the slightest reason for falling in with this piece of pathology in the present case—and indeed we have no doubt that it was one of pneumo-thorax. There is no mention made of the state of the left lung—nor does there appear to have

* The reporter says, at each *expiration*; but this is impossible. He has either made a typographical mistake, or he knows nothing of what he is talking about. EDITOR.

been any search made for an aperture in that organ. In short, we cannot compliment Mons. Reporter Portal upon the present occasion, and cannot but wonder that the Editors of our cotemporary (Journal Hebdomadaire) did not see the necessity of revising the above report.

---

